# MT-4 Suppresses Resistant Ovarian Cancer Growth through Targeting Tubulin and HSP27

**DOI:** 10.1371/journal.pone.0123819

**Published:** 2015-04-14

**Authors:** Hui Chen Pai, Sunil Kumar, Chien-Chang Shen, Jing Ping Liou, Shiow Lin Pan, Che Ming Teng

**Affiliations:** 1 Pharmacological Institute, College of Medicine, National Taiwan University, Taipei, Taiwan; 2 School of Pharmacy, College of Pharmacy, Taipei Medical University, Taipei, Taiwan; 3 National Research Institute of Chinese Medicine, Taipei, Taiwan; 4 Cancer Biology and Drug Discovery, College of Medical Science and Technology, Taipei, Taiwan; The University of Hong Kong, HONG KONG

## Abstract

**Objective:**

In this study, the anticancer mechanisms of MT-4 were examined in A2780 and multidrug-resistant NCI-ADR/res human ovarian cancer cell lines.

**Methods:**

To evaluate the activity of MT-4, we performed *in vitro* cell viability and cell cycle assays and *in vivo* xenograft assays. Immunoblotting analysis was carried out to evaluate the effect of MT-4 on ovarian cancer. Tubulin polymerization was determined using a tubulin binding assay.

**Results:**

MT-4 (2-Methoxy-5-[2-(3,4,5-trimethoxy-phenyl)-ethyl]-phenol), a derivative of moscatilin, can inhibit both sensitive A2780 and multidrug-resistant NCI-ADR/res cell growth and viability. MT-4 inhibited tubulin polymerization to induce G2/M arrest followed by caspase-mediated apoptosis. Further studies indicated that MT-4 is not a substrate of P-glycoprotein (p-gp). MT-4 also caused G2/M cell cycle arrest, accompanied by the upregulation of cyclin B, p-Thr161 Cdc2/p34, polo-like kinase 1 (PLK1), Aurora kinase B, and phospho-Ser10-histone H3 protein levels. In addition, we found that p38 MAPK pathway activation was involved in MT-4-induced apoptosis. Most importantly, MT-4 also decreased heat shock protein 27 expression and reduced its interaction with caspase-3, which inured cancer cells to chemotherapy resistance. Treatment of cells with SB203580 or overexpression of dominant negative (DN)-p38 or wild-type HSP27 reduced PARP cleavage caused by MT-4. MT-4 induced apoptosis through regulation of p38 and HSP27. Our xenograft models also show the *in vivo* efficacy of MT-4. MT-4 inhibited both A2780 and NCI-ADR/res cell growth *in vitro* and *in vivo*.

**Conclusion:**

These findings indicate that MT-4 could be a potential lead compound for the treatment of multidrug-resistant ovarian cancer.

## Introduction

Ovarian cancer is a major contributor of tumor malignancy in women [1]. Over 90% of ovarian cancer deaths are caused by drug resistance and tumor metastasis. The standard treatment consists of a combination of surgery and chemotherapy with carboplatin and paclitaxel. However, drug resistance frequently develops and leads to treatment failure. Consequently, the 5-year survival rate in advanced ovarian cancer patients is approximately 30% [2].

Drug resistance develops through several mechanisms, including pharmacokinetics, tumor microenvironment, and tumor cell-specific pathways. The most common reason for the development of drug resistance is overexpression of ATP-binding cassette (ABC) transporters such as p-glycoprotein (p-gp). P-gp, which is located at the cell membrane, binds and pumps out substrate drugs to decrease the concentration of drugs within the cells. Besides ABC transporters, other mechanisms of resistance include cell cycle alteration, drug detoxification, apoptosis impairment, and oncoprotein deregulation. For example, heat shock proteins (HSPs) can significantly increase resistance to chemotherapy [3]. HSP27 is a member of the HSP family that is responsible for cell survival under stress, such as heat shock, pH alteration, and metabolic stress. It has been shown that HSP27 increases tumor growth, metastasis, and resistance to chemotherapy in numerous types of tumors, including breast, colon, ovarian, and head and neck tumors [3–6].

Mitogen-activated protein kinases (MAPKs) are serine/threonine protein kinases that regulate both cell growth and apoptosis. For example, extracellular signal-regulated kinase (ERK) inhibitors have been shown to be successful cancer therapeutic agents. Previous research has shown that Jun N-terminal kinase (JNK) and p38 MAPK pathways are deregulated in cancer, and have positive and negative roles in modulating cell survival. Several studies have indicated that activation of p38 and JNK pathways causes apoptosis by taxol-induced mitochondrial stress. PC12 cells activate p38 and JNK kinases, and induce Fas ligand expression, which leads to apoptosis by the removal of a survival factor. P38-deficient mice are sensitized to Kras-induced lung tumorigenesis and chemically induced liver cancer [7–9].

Apoptosis is a programmed form of cell death characterized by DNA fragmentation and chromatin condensation. Caspases are essential regulators of apoptosis and can be activated through either the intrinsic (mitochondrial-mediated) or extrinsic (death receptor-mediated) apoptotic pathways. The intrinsic apoptotic pathway is characterized by the permeabilization of the mitochondria and release of cytochrome C into the cytoplasm. Cytochrome C then forms an apoptosome to initiate caspase cascade activation through caspase-9. The extrinsic pathway is activated by death receptors such as TNFR or Fas. After ligands bind to these receptors, the death-inducing signal complex is formed and initiates the caspase cascade through caspase-8 [10].

Moscatilin (4,4′-dihydroxy-3,3′,5-trimethoxybibenzyl) is one of the major bioactive compounds extracted from a traditional Chinese medicinal plant, *Dendrobium loddigesii*. Our group demonstrated that moscatilin inhibits platelet aggregation, decreases COX-2 and iNOS expression in macrophages [11], attenuates cancer growth and migration [12,13], and suppresses angiogenesis [14]. Considering the effectiveness of moscatilin, we synthesized a series of moscatilin derivatives and evaluated their biological activity both *in vitro* and *in vivo*.

## Materials and Methods

### Antibodies and chemicals

Antibodies used in this work were caspase-3, caspase-8, caspase-9, Aurora B, CDC2 Thr161, P38, GAPDH, HSP27, MKK3/6 (Cell Signaling, Beverly, MA), CDC2, PARP (Santa Cruz Biotechnology, Santa Cruz, CA), PLK, caspase-7 (BD Bioscience, San Joes, CA), Histone (Ser10), and MPM2 (Millipore, Temecula, CA). RPMI 1640 medium, fetal bovine serum (FBS), penicillin, and trypsin-EDTA were obtained from Gibco/BRL. PD98059, SB203580, SP600125, and LY294002 were from Sigma Chemicals (St. Louis, MO).

### Cell culture and transfection

The NCI/ADR-RES cell line was obtained from the DTP Human Tumor Cell Line Screen (Developmental Therapeutics Program, NCI). A2780, Hep3B, PC-3, AsPC-1, MDA-MB-231 were obtained from American Type Culture Collection (Manassas, VA, USA) and grown in RPMI 1640 supplemented with 10% heat-inactivated FBS, 100 units/mL of penicillin, and 0.1 μg/mL of streptomycin. Cells were transfected with dominant negative (DN)-p38 or wild-type HSP27 plasmids using Lipofectamine 2000 (Invitrogen), according to the manufacturer’s protocol. HSP27 plasmids were provided by Dr. Ming-Li Hsieh (Tunghai University, Taichung, Taiwan).

### Immunoblotting

To analyze the proteins, cells were lysed with iced buffer A (20 mM HEPES, pH 7.9, 2 M EGTA, 1 mM DTT, 1 μg/mL leupeptin, 5 μg/mL aprotinin, 1 mM phenylmethylsulfonyl fluoride) on ice for 30 min. Whole cell extracts were then mixed with SDS sample buffer and boiled at 95°C for 5 min. Equal amounts of proteins were subjected to SDS-PAGE and transferred to PVDF membranes. The membranes were incubated with the respective primary antibody at 4°C overnight followed by incubation with the appropriate HRP-conjugated secondary antibody. Immunoreactivity was detected with ECL substrates (Amersham).

### Co-immunoprecipitation assay

NCI-ADR/res cells were treated with 0.3 μM MT-4, 10 μM paclitaxel, or 10 μM vincristine for 48 h. Cells were disrupted in RIPA-B lysis buffer with protein inhibitors (0.5% Triton X-100, 1 μg/mL leupeptin, 5 μg/mL aprotinin, 1 mM phenylmethylsulfonyl fluoride, 0.5 M NaCl, and 0.5 M Na_2_PO_4_, pH 7.4) on ice for 30 min. After clarification by centrifugation, cell lysates were treated with 5 ng of anti-caspase-3 and incubated at 4°C overnight. For protein binding, 30 μL of protein A-G sepharose beads (Santa Cruz Biotechnology, Santa Cruz, CA) were added to each sample and incubated at 4°C for 3 h. The beads were collected and washed, and the bound proteins were eluted. Separation by SDS-PAGE was followed by western blot analysis using anti-HSP27 and anti-caspase-3 antibodies.

### Evaluation of cell viability

For the sulforhodamine B (SRB) assay, MT-4 was added to the cells for 48 h. Cells were fixed with 50% trichloroacetic acid to terminate the reaction, and 0.4% SRB in 1% acetic acid was added. After 15-min incubation, the plates were washed, and dyes were dissolved in 10 mM Tris buffer before measuring transmittance at 515 nm. For the MTT assay, following treatments, cells were incubated with 0.5 mg/mL of MTT. After 1 h, cells were lysed with DMSO before measurement of absorbance at 550 nm.

### Apoptosis assay

MT-4-induced apoptosis was assessed in A2780 and NCI-ADR/res cells using a Cell Death Detection ELISA^PLUS^ kit (Roche Diagnostics), according to the manufacturer's instructions. Growing cells were treated on the day following treatment with or without various concentrations of MT-4. After 48 h, the cell lysate solutions were placed on a streptavidin-coated 96-well plate and then incubated with monoclonal antibodies, anti-histone-biotin, and anti-DNA-POD for 2 h to bind to the histone and DNA components of the nucleosomes. After washing away unbound samples and reagents, the apoptotic oligonucleosomal fragmentation was directly detected by following the reaction between the POD moiety and the ABTS substrate and using the ELISA reader at a wavelength of 405 nm, with a reference wavelength of 490 nm.

### P-gp activity assay

NCI-ADR/res cells were pre-treated with or without the indicated compounds in culture medium in the dark at 37°C for 1 h and then co-treated with Rh-123 for an additional hour. After Rh-123 accumulation, the cells were washed with ice-cold PBS and collected by trypsinization. The intracellular fluorescence of Rh-123 was measured using an FACS flow cytometer.

### Flow cytometry

Following treatment, cells were harvested and fixed with 70% alcohol overnight at −20°C. The fixed cells were rinsed with PBS, resuspended in DNA extraction buffer (0.2 M Na_2_HPO_4_, 0.1 M citrate buffer, pH 7.8) for 30 min, and stained with PI solution containing 80 μg/mL propidium iodide, 100 μg/mL RNase, and 0.1% Tritox-100 in PBS for 30 min at room temperature. DNA content was analyzed by FACScan Flow Cytometer and CellQuest software (Becton Dickinson, USA).

### Tubulin polymerization assay

The *in vitro* microtubule polymerization assay was carried out using the cytoDYNAMIX Screen kit (Cytoskeleton Inc., Denver, CO, USA). Purified porcine tubulin was suspended in G-PEM buffer (80 mM PIPES, 2 mM MgCl_2_, 0.5 mM EGTA, 1 mM GTP, and 15% glycerol, pH 6.9) in the absence or presence of the test compound at 4°C. The mixture was transferred to pre-warmed plates before absorbance was measured at 340 nm each minute for 30 min at 37°C using a SpectraMax plus ELISA reader (Molecular Devices, Sunnyvale, CA, USA).

### Ethics statement

This study was carried out in strict accordance with the criteria outlined in the National Taiwan University “Guide for Care and Use of Laboratory Animals” for the protection of animals used for scientific purposes. Our laboratory has administrative authorization for animal experimentation, and this protocol was approved by the Institutional Animal Care and Use Committee (IACUC) of the National Taiwan University College of Medicine and College of Public Health. The IACUC approval number is 20090369. Mice were euthanized with CO_2_ inhalation, and all efforts were made to minimize animal suffering.

### *In vivo* xenograft mouse model

Six-week-old female BALB/c mice (Laboratory Animal Center, National Taiwan University) were inoculated subcutaneously (s.c.) with 5 × 10^6^ A2780 cells or 4 × 10^6^ NCI-ADR/res cells. Tumor volume was measured by caliper and calculated using the following formula: volume (mm^3^) = (width) ^2^ × length × 0.5. When the tumor volume reached 100 mm^3^, mice were randomized into groups (*n* = 5). MT-4 and paclitaxel were dissolved in a DMSO/ethanol/Cremophor (1:4:5) solution, which was then diluted with 5% glucose before treatment. MT-4 was administered intravenously (i.v.) in doses of 5 or 20 mg/kg to subjects with A2780 xenografts and in 10 mg/kg i.v. doses to subjects with NCI-ADR/res xenografts, while paclitaxel was administered in 20 mg/kg i.v. doses as positive control. Treatments were started at day 4 and stopped at day 25. Mice were humanely euthanized using CO_2_ inhalation at day 25.

### Statistical analysis

All of the *in vitro* data were expressed as the mean ± S.E.M. of at least three independent experiments and were analyzed with a Student’s *t* test. Significance was set at *P* < 0.05. A single asterisk indicates *P* < 0.05, two asterisks indicate *P* < 0.01, and three asterisks indicate *P* < 0.001. Densitometric analysis was used to show fold change of protein expression level by image J.

## Results

### MT-4 inhibits ovarian cancer growth

**Fig 1:**
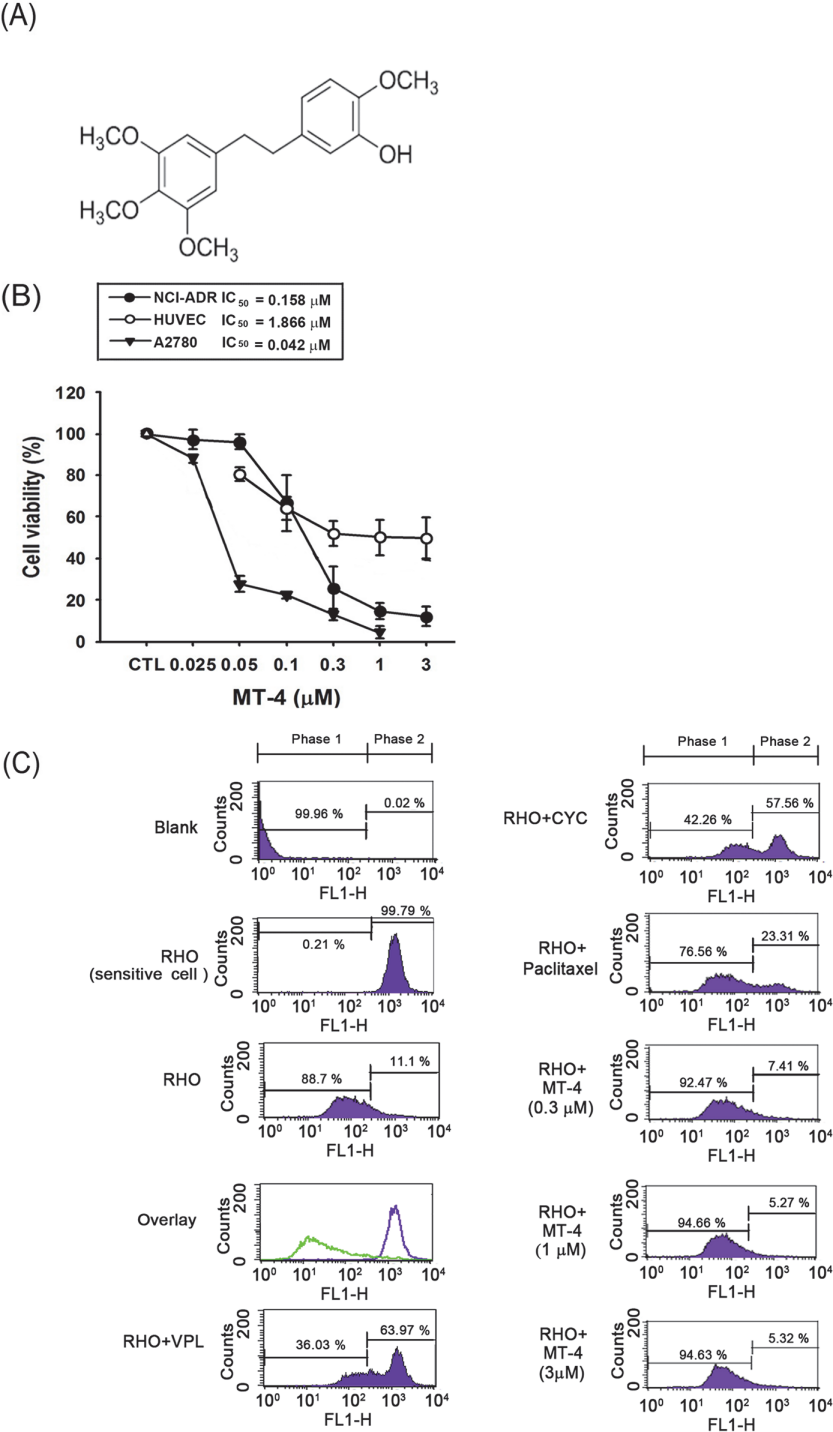
MT-4 induces apoptosis in different cancer cell lines. (A) Chemical structure of MT-4. (B) Two cancer cell lines and HUVECs were treated with MT-4 at different concentrations for cell viability. The IC_50_ of MT-4 was evaluated and compared to control by MTT assay after 48 h. The cells were incubated with MT-4 (0.025 to 3 μM) or DMSO (Control, CTL), and the absorbance was read at 570 nm. Data are presented as the mean of three replicate experiments. (C) NCI-ADR/res cells were treated with or without indicated agent (blank) and co-treated with 10 μM rhodamine 123 (RHO). After incubation for 60 min at 37°C, cells were washed with cold PBS and collected by trypsinization. Bound drugs were analyzed by flow cytometry. VPL, verapamil (30 μM); CYC, cyclosporine A (10 μM); paclitaxel (10 μM) and MT-4 (0.3, 1 and 3 μM). The sensitive cells (A2780) were as a surrogate standard to define the accumulation of Rho123 within the cells (Phase 2).

The ability of a series of moscatilin derivatives, MT-1 to MT-26 (μM), to inhibit cancer cell growth was evaluated using the SRB assay. As shown in S1 Table, we evaluated the *in vitro* anti-proliferative activities of moscatilin derivatives in six human cancer cell lines: Hep3B, PC3, AsPC-1, MDA-MB-231, A2780, and NCI-ADR/res. The GI_50_ value (μM) is the concentration of moscatilin derivatives (from 0.01 to 10 μM) to cause a 50% reduction in proliferation. Substitution of the 4′-hydroxyl group of moscatilin (4,4′-dihydroxy-3,3′,5′-trimethoxybibenzyl) with a methoxy group led to the synthesis of MT-4 (Fig 1A), which was the most effective derivative against ovarian cancer cell, A2780, with a GI_50_ of 0.04 μM. The MTT assay was performed to evaluate the effect of MT-4 on cell viability. MT-4 showed potent cytotoxicity against both A2780 and NCI-ADR/res, but much less activity against normal human umbilical vein endothelial cells (HUVECs; Fig 1B).

Interestingly, we noticed that MT-4 was effective against the multidrug-resistant ovarian cancer cell line NCI-ADR/res, compared to paclitaxel and vincristine (Table 1). The IC_50_ value of MT-4 is 17.3-fold and 66.1-fold more potent than paclitaxel and vincristine in NCI-ADR/res cells. Therefore, we next used rhodamine 123, a p-gp-transported fluorescent dye, to measure the transport activity of p-gp in NCI-ADR/res cells after MT-4 treatment. After incubation with classic p-gp inhibitors verapamil or cyclosporine A, or p-gp substrate paclitaxel, we observed significant intracellular accumulation of about 63.97%, 57.56%, and 23.31%, respectively, of rhodamine 123, which is an indicator of p-gp activity. However, the accumulation of rhodamine 123 (7.41%, 5.27% and 5.32% at 0.3, 1 and 3 μM, respectively) did not increase in NCI-ADR/res cells after incubation with MT-4 (Fig 1C). Taken together, these results suggest that MT-4 does not interfere with the function of p-gp in cancer cells.

**Table 1:** IC_50_ from MTT assay (μM).

IC_50_ (μM)	NCI-ADR/res	A2780
**MT-4**	0.158	0.0428
Paclitaxel	2.74	0.0101
Vincristine	10.45	0.018

NCI-ADR/res an A2780 were treated with MT-4, paclitaxel, and vincristine at different concentrations for cell viability. The IC_50_ was evaluated and compared to control by MTT assay after 48 h.

### MT-4 induces G2/M arrest

**Fig 2:**
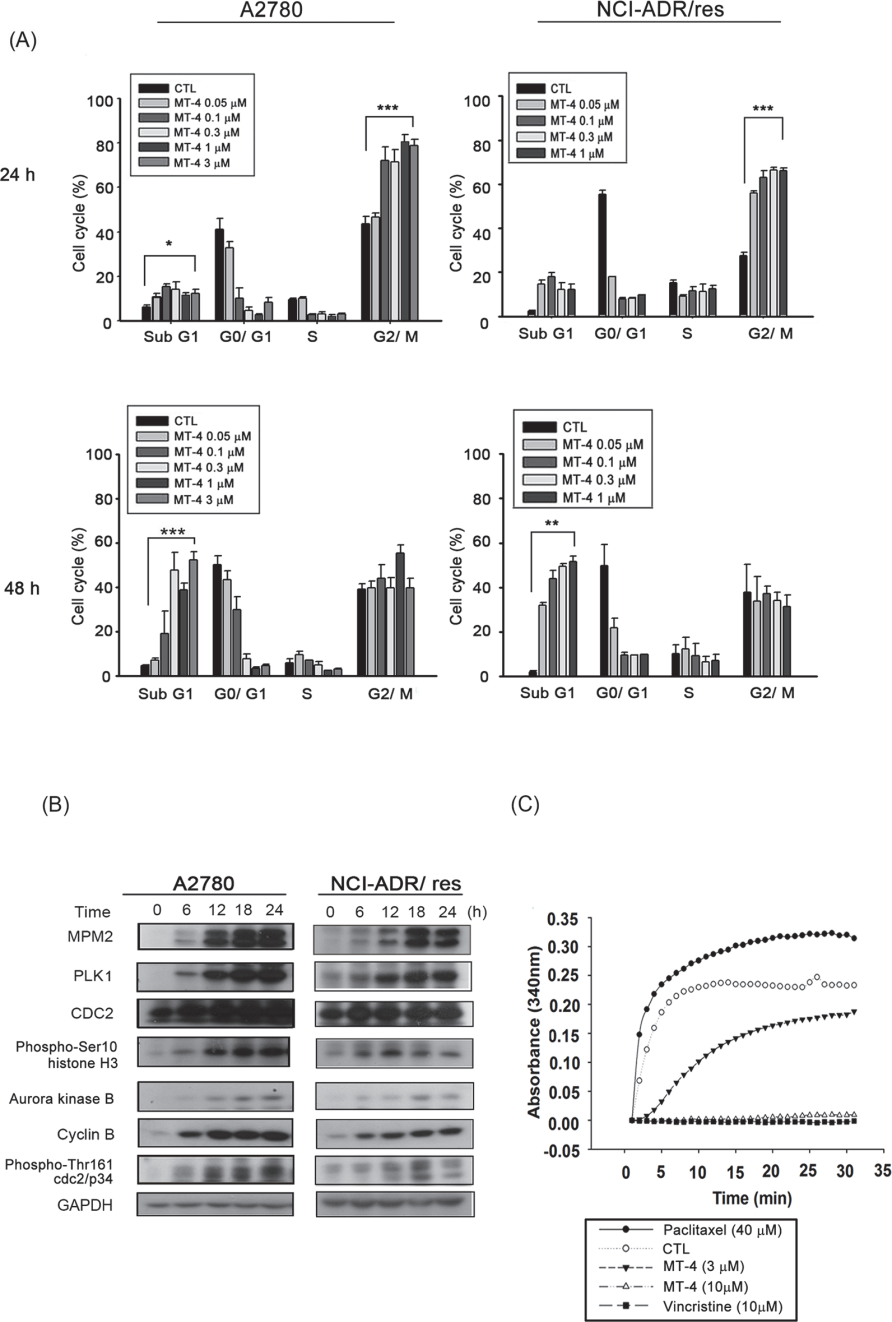
MT-4 induces G2/M arrest and regulatory proteins. (A) A2780 and NCI-ADR/res cells were treated with various concentrations of MT-4 (0.05 to 3 μM) or DMSO (Control, CTL). After incubation for 24 h and 48 h, cells were stained with propidium iodide, and cell cycles were analyzed by flow cytometry. Quantitative data are presented from three independent experiments. *p < 0.05, **p < 0.01, ***p < 0.001. (B) Both cell lines were treated with 0.3 μM MT-4 for different time intervals. Whole cell lysates were analyzed by western blot for G2/M protein. Similar results were obtained in three independent experiments. (C) Inhibition of tubulin polymerization. Tubulin in the reaction buffer was incubated at 37°C in the presence of DMSO (Control, CTL), the indicated concentration of MT-4 (3 and 10 μM), or 10 μM vincristine and 40 μM paclitaxel. The microtubule assembly was evaluated by measuring absorbance at 340 nm using a spectrophotometer.

To investigate the mechanism responsible for the growth inhibition, we examined the cell cycle distribution of MT-4-treated human cells using flow cytometry. MT-4 induced G2/M phase arrest at 24 h followed by an increase in the hypodiploid sub-G1 phase at 48 h, which indicated apoptosis (Fig 2A). To elucidate how MT-4 induced G2/M arrest, we examined the related regulatory proteins. Western blot analysis revealed that MT-4 induced a marked increase in mitosis-specific MPM2 phosphoprotein expression and upregulation of serine/threonine kinases such as polo-like kinase 1 (PLK1), Aurora kinase B, cyclin B, and phospho-Thr161-Cdc2/p34. Phosphorylation at Ser10 of Histone H3, a crucial event for chromosome condensation at the onset of mitosis, was also increased after MT-4 treatment (Fig 2B), while CDC2 had no effect. Because MT-4 caused a significant mitotic arrest and moscatilin is known to induce tubulin depolymerization, we investigated the effects of MT-4 on microtubule organization using a cell-free tubulin polymerization assay. As shown in Fig 2C, MT-4 significantly inhibited tubulin polymerization, similar to the microtubule destabilizing agent vincristine.

### MT-4 triggers apoptosis in ovarian cancer cells

**Fig 3:**
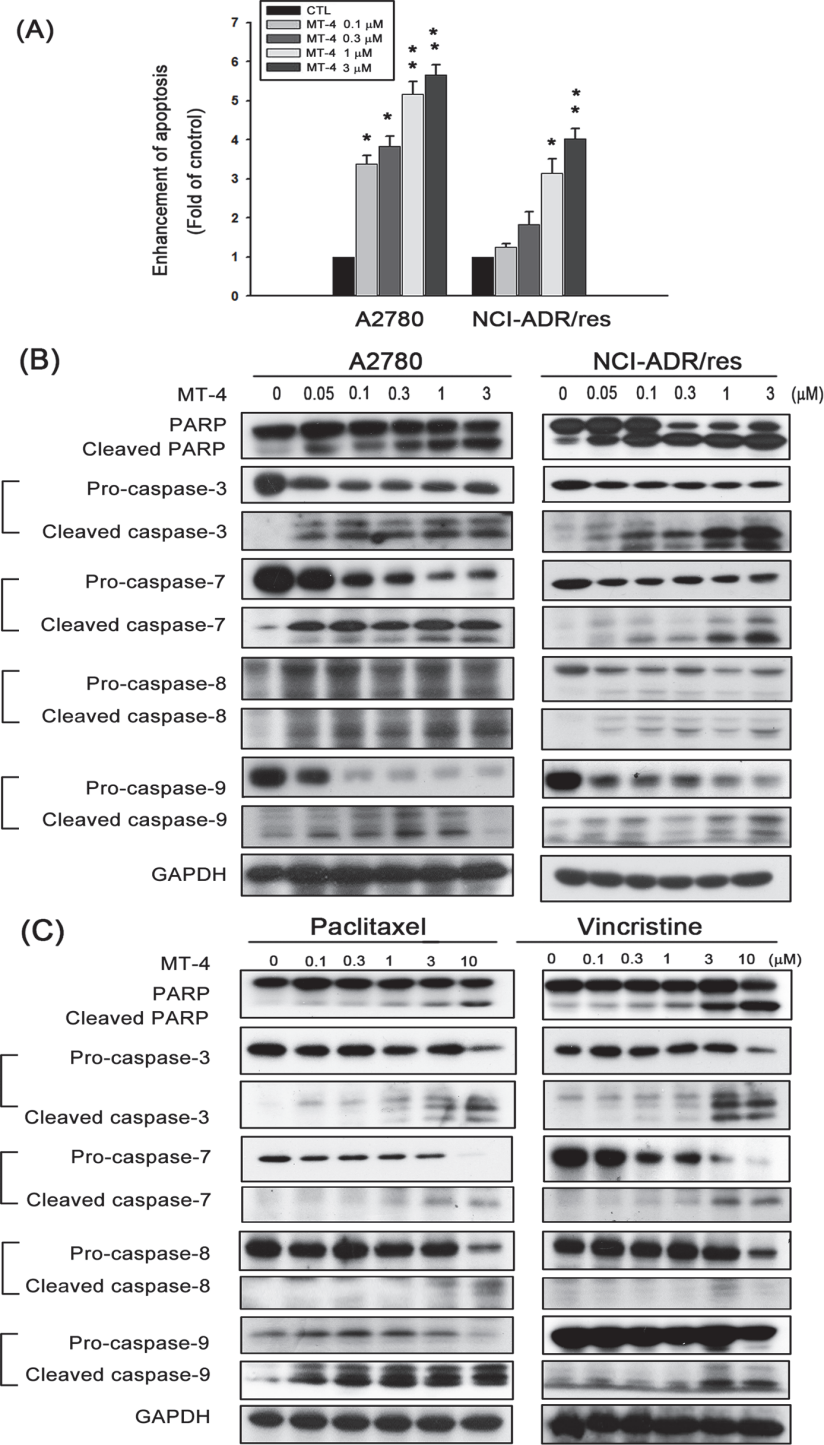
MT-4 induces apoptosis and activates caspases in an ovarian cell line. (A) The cells were treated with MT-4 at various concentrations and then analyzed using a cell death detection assay after 48 h. Quantitative data are presented from three independent experiments. *p < 0.05, **p < 0.01. (B) Both cell lines were treated with various concentrations (0.05 to 3 μM) of MT-4. After incubation for 48 h, whole cell lysates were subjected to western blot protein analysis. (C) NCI-ADR/res cells were treated with different concentrations of paclitaxel (0.1 to 10 μM) and vincristine (0.1 to 10 μM). After incubation for 48 h, the cell lysates were subjected to western blot analysis. Similar results were obtained in three independent experiments.

Induction of apoptosis by MT-4 was indicated by the appearance of a subG1 population in ovarian cancer cells, as confirmed by measuring cytoplasmic histone-associated DNA fragments using a cell death detection ELISA kit. DNA fragmentation increased after MT-4 treatment in a concentration-dependent manner (Fig 3A). Further evidence of MT-4-induced apoptosis was provided by experiments showing MT-4-induced activation of caspase-3, -7, -8, and -9, and cleavage of PARP (Fig 3B). Compared to MT-4, only 10 μM paclitaxel and 3μM vincristine can induce apoptosis in NCI-ADR/res cells, suggesting that MT-4 is more effective than these two drugs in current clinical use in multidrug-resistant cells (Fig 3C).

### Activation of the p38 MAPK pathway contributes to MT-4-induced apoptosis

**Fig 4:**
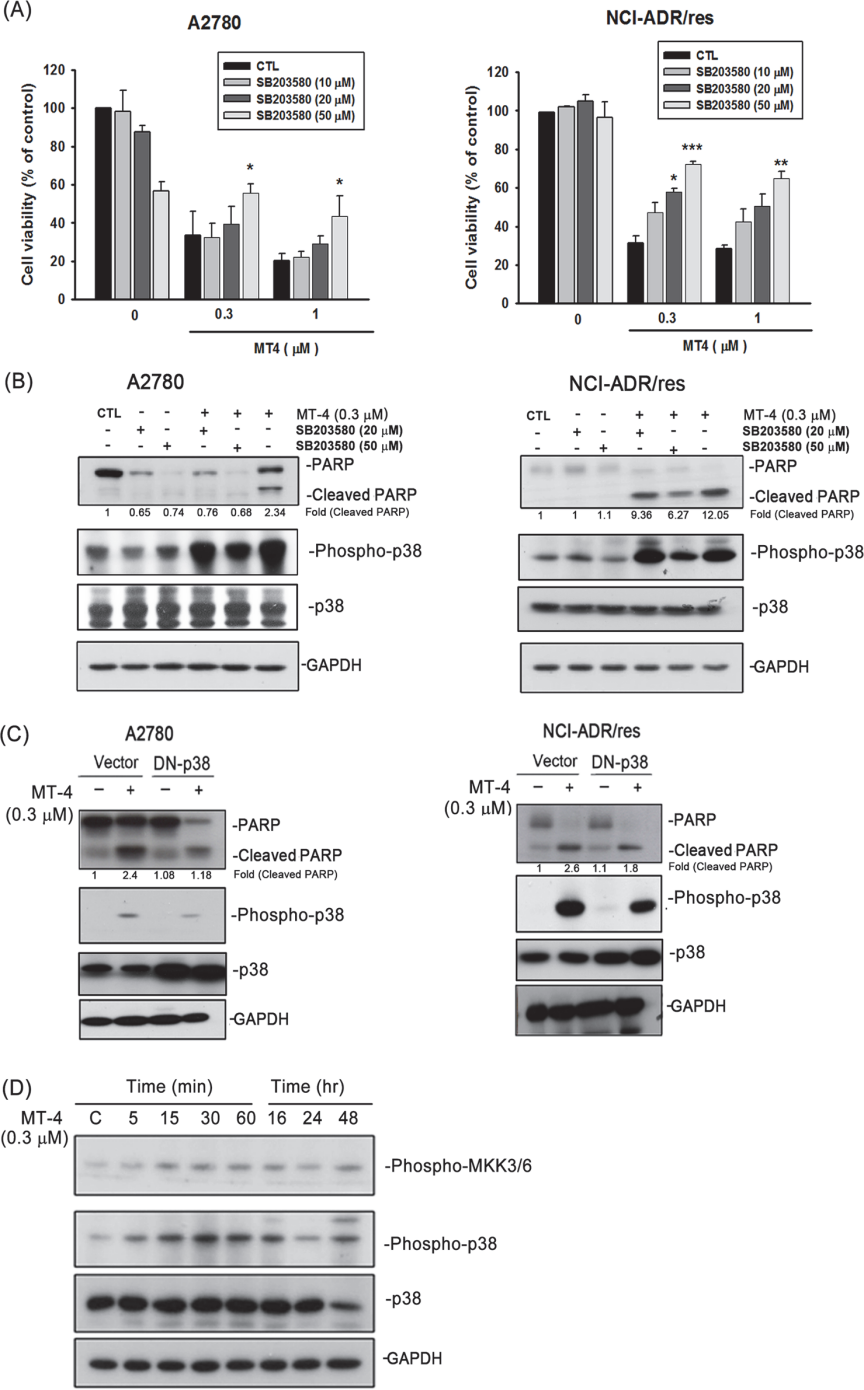
Activation of p38 MAPK contributes to MT-4-induced apoptosis. (A) A2780 and NCI-ADR/res cells were treated with MT-4 (0.3 and 1 μM) or DMSO (Control, CTL) or in the presence of SB203580 (10, 20, and 50 μM). After incubation for 48 h, viable cells were analyzed by MTT assay. *p < 0.05, **p < 0.01, ***p < 0.001. Data represent the mean ± S.E.M. of at least three independent experiments. (B) The cells were treated with DMSO (Control, CTL) and 0.3 μM MT-4 alone, SB203580 alone (20 μM or 50 μM), or a combination of both reagents for 48 h. Cell apoptosis via PARP and p38 protein was evaluated by western blot. (C) Both cells were transfected with or without 2 μg of dominant-negative (DN)-p38 plasmid and then incubated with MT-4 (0.3 μM) for 48h. Whole cell lysates were subjected to western blot analysis. (D) NCI-ADR/res cells were treated with 0.3 μM MT-4 for different time intervals. Whole cell lysates were subjected to western blot analysis for MKK3/6 and p38 protein. Similar results were obtained in three independent experiments.

Previous studies have indicated that moscatilin induces apoptosis through JNK activation [12], attenuates cell motility through AKT inhibition [13], and decreases angiogenesis through both ERK and AKT suppression [14]. To further characterize the upstream signals involved in the induction of apoptosis in MT-4-treated ovarian cancer cells, inhibitors of PI3K and MAPK, including LY294002, PD98059, SB203580, and SP600125, were therefore tested for their capacity to ameliorate apoptosis (Fig 4A and S1 Fig). Only the p38 inhibitor, SB203580, significantly reversed MT-4-induced cell death. MT-4 activated phospho-p38 and induced PARP cleavage, and these functions could be reversed by treatment with SB203580 in a concentration-dependent manner (Fig 4B) or overexpression of dominant negative (DN)-p38 (Fig 4C). With regard to the importance of p38 activation for the susceptibility of ovarian cancer to MT-4, it was of interest to observe the activation of p38 after MT-4 treatment. MT-4 indeed induced MKK3/6-p38 phosphorylation (Fig 4D), consistent with a role for p38 activation in MT-4-induced apoptosis.

### MT-4 decrease HSP27 expression through p38 activation

**Fig 5:**
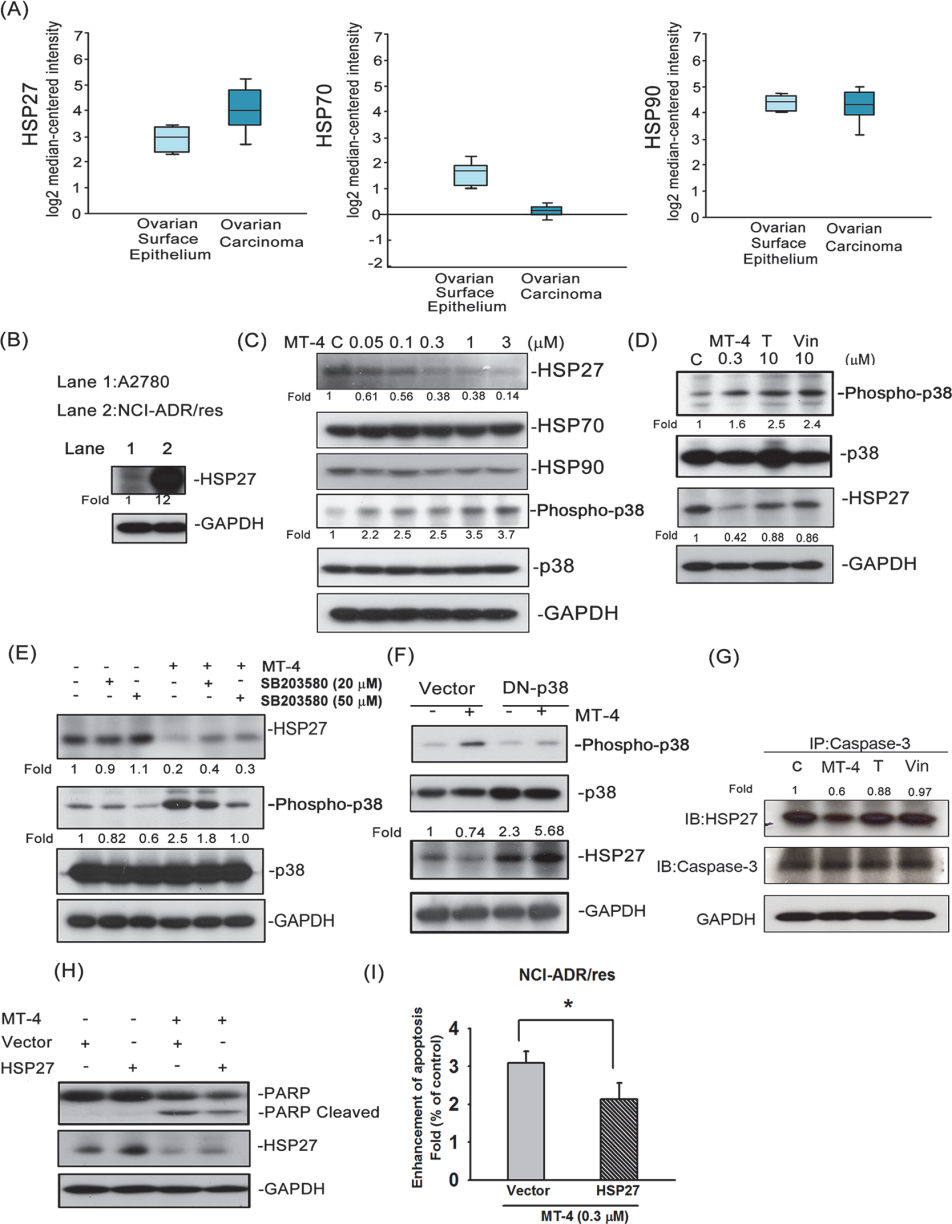
MT-4 regulated HSP27 protein expression. (A) HSP27, HSP70, and HSP90 mRNAs were analyzed from the Bonome dataset. (B) A2780 and NCI-ADR/res cells were lysed and analyzed by western blot for HSP27 protein. Lane 1, A2780 and Lane 2, NCI-ADR/res. (C) NCI-ADR-res cells were treated with the indicated concentrations of MT-4 (0.05 to 3 μM) and DMSO (Control, C) for 48 h. (D) NCI-ADR/res cells were treated with 0.3 μM MT-4, 10 μM paclitaxel (T), or 10 μM vincristine (Vin) or DMSO (Control, C) for 48 h. (E) NCI-ADR/res cells were treated with 0.3 μM MT-4, SB203580 alone (20 μM or 50 μM) or a combination of both reagents for 48 h. Whole cell lysates were evaluated using western blot analysis for the indicated proteins. (F) NCI-ADR/res cells were transfected with or without 2 μg of domain-negative p38 plasmid and then incubated with MT-4 (0.3 μM) for 48 h. Whole cell lysates were subjected to western blot analysis. Similar results were obtained in at least three other independent experiments. (G) Co-immunoprecipitation (Co-IP) assay and western blot assay. NCI-ADR/res cells were treated as indicated, and lysates were subjected to a Co-IP assay with anti-caspase-3 and were blotted with anti-HSP27 and anti-caspase-3. (H) The cells were transfected with or without 5 μg of wild-type HSP27 plasmid and then incubated with MT-4 (0.3 μM) for 48 h. Whole cell lysates were subjected to western blot analysis and (I) cell death detection kit assays. *p < 0.05. The data represent the mean ± S.E.M. of at least three independent experiments. Densitometric analysis shows fold change in protein levels compared with control group by image J.

Expression of HSP27, but not HSP70 or HSP90, in ovarian cancer has been suggested as a reliable indicator of prognosis [15]. We compared mRNA levels of Hsp27, Hsp70, and Hsp90 from the Bonome dataset [16] containing 10 normal ovarian surface epithelium and 185 ovarian carcinoma samples using the Oncomine database (www.oncomine.org). Only HSP27 expression was significantly higher in ovarian cancer (Fig 5A), which implies that HSP27 may be involved in neoplastic transformation. Considering the presumed oncogenic role of HSP27 and that the expression of HSP27 induces resistance to anticancer drugs such as paclitaxel [17], we compared the expression of HSP27 among different ovarian cancer cell lines. As shown in Fig 5B, the expression of HSP27 was about 12-fold higher in NCI-ADR/res cells. MT-4 significantly inhibited HSP27 expression and increased phosphorylation of p38 in a concentration-dependent manner (Fig 5C), compared to vincristine and paclitaxel (Fig 5D). Notably, the MT-4-mediated downregulation of HSP27 and activation of p38 were partially attenuated by SB203580 (Fig 5E) or overexpression of DN-p38 (Fig 5F), which indicates that p38 activation is involved in HSP27 expression mediated by MT-4 inhibition. HSP27 has been reported to interact with procaspase-3 to prevent the formation and/or function of the apoptosome complex [18]. With regard to MT-4 inhibition of HSP27 expression and increase in caspase-3 activation, it was of interest to observe the association of HSP27 and caspase-3 after MT-4 treatment. As shown in Fig 5G, MT-4 inhibited HSP27 protein expression levels, which resulted in decreased interaction with caspase-3. Caspase-3, unbound from HSP27, activated an increased apoptotic rate in NCI-ADR/res cells. We used overexpression of the HSP27 plasmid transfected into cells to determine whether HSP27 is involved in MT-4-induced apoptosis and to demonstrate whether HSP27 downregulation by MT-4 is involved in apoptotic effects. As shown in Fig 5H and 5I, MT-4 induced PARP cleavage and increased DNA fragmentation in NCI-ADR/res cells, as detected by western blot and cell death detection ELISA kit, respectively. We found that MT-4-induced PARP and DNA fragments were abolished by co-treatment with overexpressed HSP27 plasmids, suggesting that MT-4-induced HSP27 downregulation is one of the causes of apoptosis.

### MT-4 inhibits ovarian cancer growth *in vivo*

**Fig 6:**
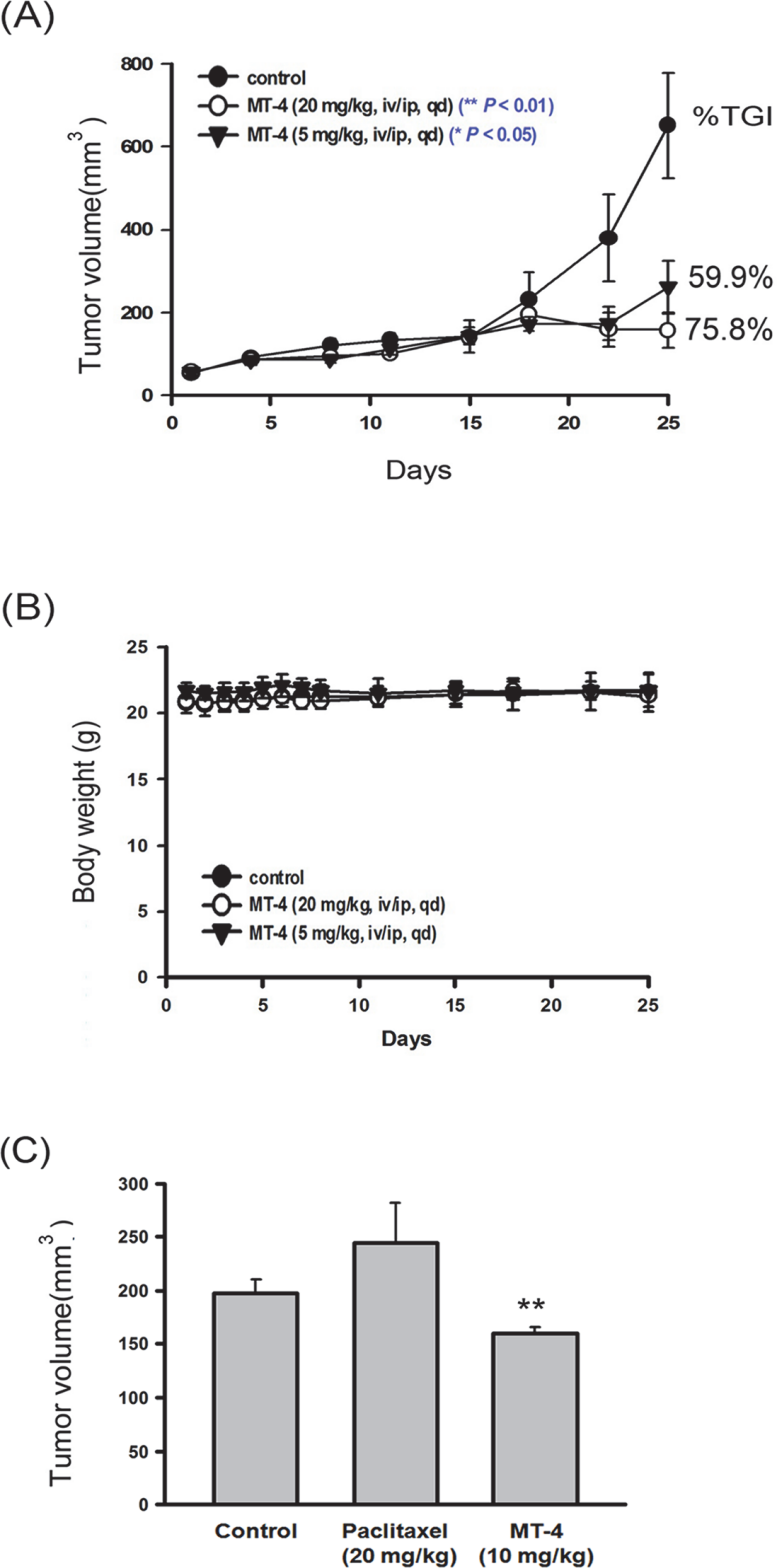
MT-4 triggers apoptosis in an ovarian xenograft animal model. Xenograft nude mice were intravenously (i.v.) and intraperitoneally (i.p.) administered MT-4 (5 mg/kg and/or 20 mg/kg) daily, paclitaxel (20 mg/kg, i.v.) every 4 days for a total of 5 days, or a vehicle after tumor cell (A2780 and NCI-ADR/res) implantation. (A-B) Tumor size and body weight of the A2780 xenograft mice. (C) Tumor size of the NCI-ADR/res xenografts. * *p* < 0.05, **p < 0.01 compared to controls.

Finally, we determined the *in vivo* anti-tumor activity of MT-4 using a xenograft model. Treatment of A2780 xenograft-bearing mice with MT-4 resulted in significant tumor growth delay (TGD; 59.9% for 5 mg/kg, 75.8% for 20 mg/kg; Fig 6A) without loss of body weight (Fig 6B), suggesting that MT-4 is an effective anticancer agent at dosages that induce negligible side effects. More importantly, we found that MT-4 (10 mg/kg) but not paclitaxel (20 mg/kg) inhibits multidrug-resistant NCI-ADR/res growth in mice (Fig 6C), which supports that MT-4 can suppress resistant ovarian cancer growth *in vivo*. MT-4 not only caused tumor growth delay in sensitive A2780 mice but also partially inhibited tumor growth in multidrug-resistant NCI-ADR/res mice.

## Discussion

As much as 75% of patients with ovarian cancer eventually relapse within 18 months, and many of them develop resistance to chemotherapy. In our research, we demonstrated that a novel moscatilin analog, MT-4, overcomes p-gp-mediated drug resistance to promote cell death in resistant NCI-ADR/res cells at a lower concentration than current anticancer drugs such as paclitaxel and vincristine. In addition, MT-4 induces apoptosis through a caspase-dependent pathway initiated by tubulin destabilization and p38 activation in ovarian cancer cells *in vitro* and *in vivo*.

MT-4 was significantly more effective than moscatilin against ovarian cancer (A2780: 173.5-fold, NCI/ADR: 21.9-fold). Similar to moscatilin, MT-4 induced tubulin depolymerization followed by mitotic arrest and apoptosis. Moscatilin has been demonstrated to induce colon cancer apoptosis through JNK activation [12], inhibit breast cancer motility through AKT inhibition [13], and decrease angiogenesis through both ERK and AKT suppression [14]. Consistent with previous studies, we determined the role of MAPK signaling in MT-4-induced ovarian cancer apoptosis. We found that MT-4 only activated p38 to decrease the expression of HSP27, subsequently promoting caspase-3-mediated apoptosis. After chemical modification, the anticancer effect of MT-4 is more potent than moscatilin in ovarian cancer cells, especially in resistant cells. Many of the p38-MAPK stress pathway stimuli, such as heat shock, TNF-α, and irradiation, can directly activate and phosphorylate p38 at threonine/tyrosine sites by MKK3 and MKK6 [7]. This pathway controls transcription factors and downstream kinases, which can modulate cell fate. Our results showed that MT-4 induced phosphorylation of MKK3/6 and the p38 pathway in a time-dependent modulation of cellular apoptosis. SB203580 (a p38 inhibitor) could reverse MT-4-induced cell apoptosis in ovarian cancer cells. Moreover, cells transfected with DN-p38 plasmids showed a decrease in the cleaved form of PARP after treatment with MT-4.

Based on these data, we suggest that activation of p38-MAPK via MKK3/6 pathways promoted MT-4-induced apoptosis in both ovarian cancer cell lines investigated. In previous studies, p38 activation has been shown to regulate phosphorylation of HSP27. Subsequently, phosphorylated HSP27 results in structural changes and promotes the transition of the HSP27 protein from an oligomer to a monomer or dimer, which can escape degradation by proteases [19,20]. In addition, phosphorylation of HSP27 has been reported to suppress apoptosis and develop drug resistance. However, our results demonstrated that MT-4 activated phosphorylation of p38 and induced apoptosis but decreased phosphorylation of HSP27 (data not shown), which implies that there may be other regulators involved in this interaction. Furthermore, HSP27 has been reported to directly bind to tubulin with paclitaxel [21]. We suggest that MT-4-induced tubulin depolymerization might disrupt the interaction between HSP27 and tubulin and activate p38 at the same time. However, more evidence is required to identify a correlation between p38 and HSP27 signaling after MT-4 treatment.

HSP27 is involved in chemotherapy resistance in ovarian cancer. Cells transfected with the HSP27 antisense gene were more sensitive to cisplatin [22,23]. Our work demonstrated that MT-4 inhibits HSP27 but did not alter HSP70 and HSP90 expression. Although it is still not clear how MT-4 inhibits HSP27 expression, decreasing HSP27 expression can decrease drug resistance. For example, paclitaxel could inhibit HSP27 to overcome drug resistance in ovarian and uterine cancer cells [24]. In our study, MT-4 was shown to have better inhibitory effects on HSP27 protein levels compared with paclitaxel and vincristine. HSP27 is known to prevent proteolytic processing of caspase-3 through association with its prodomain [25,26]. Experiments were therefore performed to examine whether HSP27/casapse-3 association was affected in MT-4-treated cells. Because MT-4 inhibits the expression of HSP27, procaspase-3 was released from HSP27 to initiate apoptosis. These data provide one of the mechanisms by which HSP27 contributes to MT-4-induced apoptosis. Based on these results, we suggested that MT-4 induced the resistant NCI-ADR/res cell apoptosis via regulation of HSP27 protein expression and function. This suggests that HSP27 may play an important role in the treatment of resistant cancers.

MT-4, a derivative of moscatilin, exhibited a significantly increased apoptosis rate in both sensitive and resistant human ovarian cancer cells *in vitro* and promoted tumor growth delay in an animal model. MT-4 promotes tubulin depolymerization and cell apoptosis via p38 and HSP27 pathways. In conclusion, we demonstrated that MT-4 represents a novel and potent anticancer agent against ovarian cancer cells, especially in resistant cells.

## Supporting Information

S1 Fig Effect of PI3K and MAPK inhibitor on MT-4-induced apoptosis in ovarian cancer cells. A2780 and NCI-ADR/res cells were treated with MT-4 (0.3 and 1 μM) or (A) PD98059 (10 and 20 μM), (B) SP600125 (17.5 and 25 μM), and (C) LY294002 (10 and 20 μM). After incubation for 48 h, viable cells were analyzed by MTT assay.
(TIF)

S1 Table Moscatilin derivatives inhibit cell growth in different cells. The cells include human hepatoma (Hep 3B), prostate cancer (PC-3), human pancreatic adenocarcinoma (AsPC-1), breast cancer (MDA-MB-231), and ovarian cancer (A2780 and NCI-ADR/res) cell lines. Cancer cells were treated for 48 h with different concentrations (0.01 to 10 μM) of moscatilin derivatives (MT-1 to MT-26) and measured by SRB assay.
(DOCX)
