# Supplementary figures and images for: MT-4 Suppresses Resistant Ovarian Cancer Growth through Targeting Tubulin and HSP27
Source: PLoS One. 2015 Apr 14;10(4):e0123819. doi: 10.1371/journal.pone.0123819 (PMC4397017; doi:10.1371/journal.pone.0123819)

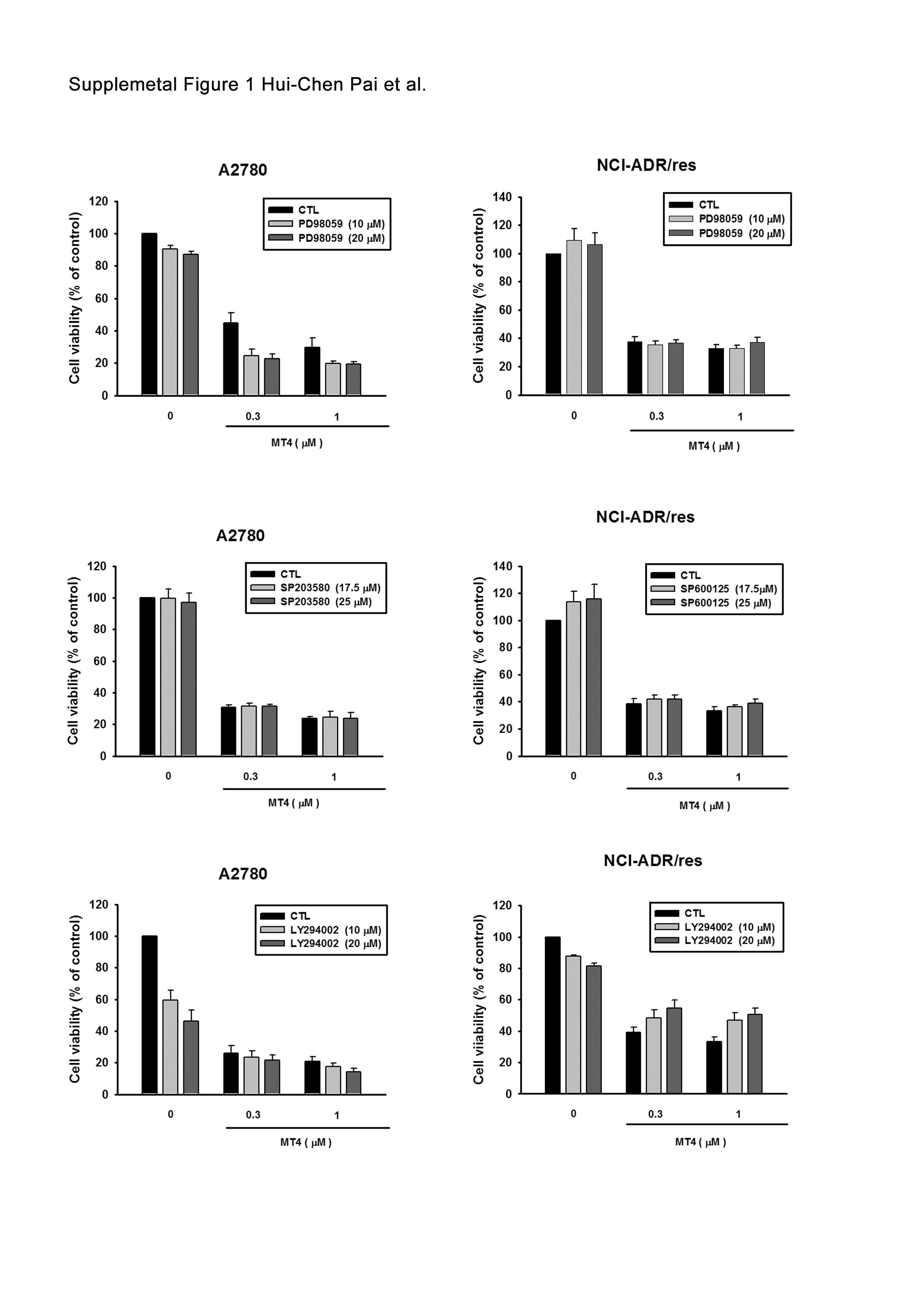

Supplement: S1 Fig — A2780 and NCI-ADR/res cells were treated with MT-4 (0.3 and 1 μM) or (A) PD98059 (10 and 20 μM), (B) SP600125 (17.5 and 25 μM), and (C) LY294002 (10 and 20 μM). After incubation for 48 h, viable cells were analyzed by MTT assay. (TIF) [file pone.0123819.s001.tif]
