# Supplementary material for: MT-4 Suppresses Resistant Ovarian Cancer Growth through Targeting Tubulin and HSP27
Source: PLoS One. 2015 Apr 14;10(4):e0123819. doi: 10.1371/journal.pone.0123819 (PMC4397017; doi:10.1371/journal.pone.0123819)
Supplement: S1 Table — The cells include human hepatoma (Hep 3B), prostate cancer (PC-3), human pancreatic adenocarcinoma (AsPC-1), breast cancer (MDA-MB-231), and ovarian cancer (A2780 and NCI-ADR/res) cell lines. Cancer cells were treated for 48 h with different concentrations (0.01 to 10 μM) of moscatilin derivatives (MT-1 to MT-26) and measured by SRB assay. (DOCX) [file pone.0123819.s002.docx]

**Supporting Tables**

**S1 Table. Moscatilin derivatives inhibit cell growth in different cells.**

| **GI50(μM)** | **Hep-3B** | **PC-3** | **AsPC-1** | **MDA-MB-231** | **A2780** | **NCI-ADR/res** |
| --- | --- | --- | --- | --- | --- | --- |
| MT-01 | 7.04 | ＞10 | ＞10 | 3.22 | 4.38 | 4.49 |
| MT-02 | ＞10 | ＞10 | ＞10 | 8.72 | ＞10 | ＞10 |
| MT-03 | ＞10 | 23.57 | ＞30 | ＞10 | ＞10 | ＞10 |
| MT-04 | 0.25 | 0.1 | ＞30 | 0.03 | 0.04 | 0.14 |
| MT-05 | ＞10 | ＞10 | ＞10 | ＞10 | ＞10 | ＞10 |
| MT-06 | 0.11 | 0.06 | 0.09 | 0.06 | 0.059 | 0.055 |
| MT-07 | ＞10 | ＞10 | ＞10 | ＞10 | ＞10 | ＞10 |
| MT-08 | ＞30 | ＞30 | ＞10 | ＞10 | ＞10 | ＞10 |
| MT-09 | 1.19 | 0.02 | 1.78 | 0.02 | 0.04 | 0.032 |
| MT-10 | ＞30 | ＞30 | ＞10 | ＞10 | ＞10 | ＞10 |
| MT-11 | ＞10 | ＞10 | ＞10 | ＞30 | ＞10 | ＞10 |
| MT-12 | 0.5 | 0.05 | ＞30 | 0.02 | 0.07 | 0.028 |
| MT-13 | ＞10 | ＞10 | ＞10 | ＞10 | ＞10 | ＞10 |
| MT-14 | ＞10 | ＞10 | ＞10 | ＞10 | ＞10 | ＞10 |
| MT-15 | ＞10 | ＞10 | ＞10 | ＞10 | ＞10 | ＞10 |
| MT-16 | 2.11 | 0.6 | 0.84 | 0.58 | 0.64 | 0.25 |
| MT-17 | 2.76 | 0.24 | 0.28 | 0.25 | 0.32 | 0.21 |
| MT-18 | ＞10 | ＞10 | ＞10 | ＞10 | ＞10 | ＞10 |
| MT-19 | ＞10 | ＞10 | ＞10 | ＞30 | ＞10 | ＞10 |
| MT-20 | 4.29 | 1.03 | ＞10 | 0.67 | 1.51 | 1.29 |
| MT-21 | 1.77 | 0.86 | ＞30 | 0.70 | 1.22 | 0.99 |
| MT-22 | 1.25 | 0.64 | 1.37 | 0.50 | 0.71 | 0.50 |
| MT-23 | 3.16 | 1.53 | ＞30 | 0.86 | 0.56 | 6.89 |
| MT-24 | 0.23 | 0.19 | ＞30 | 0.08 | 0.12 | 0.10 |
| MT-25 | 1.55 | 0.07 | 8.07 | 0.60 | 0.07 | 0.43 |
| MT-26 | 3.27 | 0.86 | 0.24 | 0.07 | 0.12 | 0.04 |
| Moscatilin | 3.29 | 2.69 | ＞30 | 4.46 | 6.94 | 3.07 |
